# Technical efficiency of neonatal health services in primary health care facilities of Southwest Ethiopia: a two-stage data envelopment analysis

**DOI:** 10.1186/s13561-019-0245-7

**Published:** 2019-10-27

**Authors:** Kiddus Yitbarek, Gelila Abraham, Ayinengida Adamu, Gebeyehu Tsega, Melkamu Berhane, Sarah Hurlburt, Carlyn Mann, Mirkuzie Woldie

**Affiliations:** 1 Department of Health Policy and Management, Institute of Health, Jimma University, Jimma, Ethiopia; 2 Department of Public health, Bahirdar University, Bahirdar, Ethiopia; 3 Department of Pediatrics and child health, Institute of health, Jimma University, Jimma, Ethiopia; 4 Department of Global Health and Population, Harvard T.H. Chan School of Public Health, Boston, USA

**Keywords:** Technical efficiency, Data envelopment analysis, Neonatal health services, Primary health care units, Southwest Ethiopia

## Abstract

**Background:**

Disparity in resource allocation is an issue among various health delivery units in Ethiopia. To sufficiently address this problem decision-makers require evidence on efficient allocation of resources. Therefore, the purpose of this study was to assess the technical efficiency of primary health care units providing neonatal health services in Southwest Ethiopia.

**Methods:**

Two-stage data envelopment analysis was conducted based on one-year (2016/17) data from 68 health posts and 23 health centers in Southwest Ethiopia. Primary data were collected from each of the facility, respective district health offices and finance and economic cooperation offices. Technical efficiency scores were calculated using data envelopment analysis software version 2.1. Tobit regression was then applied to identify determinants of technical efficiency. STATA version 14 was used in the regression model and for descriptive statistics.

**Results:**

By utilizing the best combination of inputs, eight health posts (11.76%) and eight health centers (34.78%) were found to be technically efficient in delivering neonatal health services. Compared with others included in the analysis, inefficient health delivery units were using more human and non-salary recurrent resources. The regression model indicated that there was a positive association between efficiency and the health center head’s years of experience and the facility’s catchment population. Waiting time at the health posts was found to negatively affect efficiency.

**Conclusions:**

Most of health posts and the majority of health centers were found to be technically inefficient in delivering neonatal health services. This indicates issues with the performance of these facilities with regards to the utilization of inputs to produce the current outputs. The existing resources could be used to serve additional neonates in the facilities.

## Introduction

Globally, major progress has been made in improving child survival. The under-five mortality rate has declined by almost half since 1990, dropping from 90 to 46 deaths per 1000 live births in 2013 [1]. As global rates of under-five mortality have fallen, neonatal deaths now account for a rising proportion of the remaining burden of under-five deaths. Sub-Saharan Africa (SSA) remains the region with the highest under-five mortality rate in the world. There, one child in 12 dies before his or her fifth birthday; in high-income countries, the ratio is 1 in 147 [2, 3].

The first 28 days of life – the neonatal period – is the most vulnerable time for a child’s survival [4]. The proportion of under-five deaths during the neonatal period has increased despite progress in reducing neonatal mortality worldwide in the last 25 years. In 1990, neonatal deaths represented 40% of global under-five deaths, compared with 45% today. Of the estimated 5.9 million child deaths in 2015, almost 1 million occurred in the first day of life and close to 2 million took place in the first week. This is an urgent call for an increasing focus on newborns [2, 5].

In Ethiopia, neonatal mortality is a big problem which accounts for the lion’s share of under-five morality. Like the trends in other countries, the highest contributing age group for under-five mortality is the first 28 days from birth. In Ethiopia nearly half (43.3%) of under-five deaths are due to deaths in the neonatal period. This is far higher than the average of 35% for SSA. Therefore, neonatal health must be prioritized to sustain the rapid progress in reduction of overall child mortality [1]. The Sustainable Development Goals (SDGs) aim to end preventable deaths of newborns and children under 5 years of age by 2030, with all countries aiming to reduce neonatal mortality to at least as low as 12 per 1000 live births [6].

To handle the health service needs of the community health facilities are expected to deliver appropriate service packages to improve health outcomes of neonates [7]. As a result, health service planners and resource allocators have to be well informed about how to use scarce resources to save more lives. Technical efficiency analysis of health service delivery enables health service decision-makers to allocate available scarce resources in a wise manner in various settings [8–10]. However, many health resource allocators are not currently in a position to use these method for resource allocation because this type of analysis in not routine to the system. As a result, uneven distribution of health service resources relative to use is an observable condition in Ethiopia.

In addition to uneven allocation of resources relative to use in the Ethiopian health care system, poor quality neonatal health service fuel poor health status and mortality of newborns [11, 12]. Policy and decision-makers in the health system ought to use decision models in order to allocate the available scarce resources more appropriately between different health service delivery units to maximize efficiency [13]. Therefore, this study aimed to assess the technical efficiency of neonatal health service delivered in the Ethiopian primary health care system.

Technical efficiency studies were previously conducted in south west and northern Ethiopia [14, 15]. One previous study addressed technical efficiency of health centers, while another one analyzed health posts. However, both studies were not specific to neonatal care and have assessed the overall technical efficiency. This study was intended to measure the efficiency gaps in neonatal health service. Moreover, our study focuses on where the majority of primary health care is utilized - health centers and health posts under the supervision of health centers.

## Methods

### Study design and setting

A cross-sectional study was conducted in health centers and health posts located in eight districts of Jimma Zone, southwest Ethiopia, from 19 March to 28 April, 2018. Jimma zone is divided into 20 districts and one town administration with a total of 548 kebeles (the smallest administrative unit) among which 515 are rural. Based on the 2007 Census, the projected total population of the zone was 3,209,127 in 2017 [16]. In the zone there are 5 primary hospitals, 115 health centers and 520 health posts.

### Variables

#### Input variables

Input variables for health centers were non-salary recurrent expenses (expenses for vaccine, drug and supplies), administrative staff, clinical and midwife nurses, laboratory technicians and technologists, pharmacy technicians and pharmacists, and health officers. On the other hand, non-salary recurrent expenses and health extension workers were input variables for health posts. The non-salary recurrent expenditure includes expenses for vaccine, medicine and supplies.

#### Output variables

In health centers, the number of neonatal outpatients, neonatal referral and those who received service at maternal and child health units were the outputs. For health posts, we used neonatal outpatients, neonatal referrals to health centers and home-to-home service as outputs.

#### Tobit regression

The dependent variable was the technical efficiency scores. The explanatory variables used in the model were service years of the facility, facility head’s years of experience, size of the catchment population, availability of health facility around (availability of health facility with in the town, if it is urban setting and availability of health facility in the *kebele* if the setting is rural), facility head’s level of education, neonates in the catchment population and waiting time for service.

### Data collection

Prior to data collection health facilities for the study were identified as per the recommendation in the Tools for Assessing the Operationality of District Health Systems [17]; eight districts (40%) were included in the study from the total 20 districts in Jimma Zone. Three health centers from each of the districts were assessed (a total of 24). Similarly, three health posts under the supervision of each health center were included (a total of 72).

Data collection tools were developed after analyzing community based newborn care (CBNC), integrated management of newborn and child illness (IMNCI) and neonatal intensive care unit (NICU) guidelines [4, 18, 19] and other relevant literatures [15, 20, 21]. Resource inventory and document review checklists were used to collect data on the necessary resources available and/or used for neonatal health service packages. Data collection tools were first prepared in English then translated into Afaan Oromo, since the working language in the facilities is Afaan Oromo, then back translated into English by an independent translator to check for consistency.

The tools included questions on input and output variables, and organizational and environmental factors that may affect the technical efficiency of health facilities. We have collected input and output data for the Ethiopian fiscal year 2009 (July 2016 to June 2017). Nine trained data collectors and two supervisors have collected relevant data for this study.

### Source of cost data and costing

Both the financial and economic costs were considered in this study. We have visited all the study health facilities to collect cost information from their records and with resource inventory. We then get back to district health office and finance and economic cooperation office to verify the information obtained from each facility.

Costing was carried out from the provider’s perspective (provider-related costs incurred only on delivery of the interventions) that are incurred at the selected government health facilities in Jimma Zone from July 2016 to June 2017 (Ethiopian one fiscal year). All costs were estimated to the values using the current local market price. The cost of donated resources was estimated using the current market price [22].

In this evaluation ingredient (resource cost method) approach was used to calculate program costs. Bottom up for human resource salary, medication and supplies. This approach includes itemizing the resources necessary to provide neonatal health services, and calculating or estimating the costs of each resource. Those information collected in Ethiopian birr (ETB) were changed into United States dollar (US$) using the May, 2018 exchange rate of birr to dollar 27.3755 [23].

The inputs used in facilities were not directed to neonatal health service only. Almost all of the resources were allocated to the entire health facility. So, to have specific figures for neonatal health service a proportion for each health facility was computed by dividing the total volume of neonatal health services delivered by the overall client flow of each facility. All the resources then multiplied by the proportion and the costs of neonatal health service resources were determined.

### Data processing and analysis

The collected data were checked for completeness, edited and entered in to EpiData version 3.1, then exported to Microsoft office excel and STATA version 14 for analysis. Descriptive information of inputs and outputs was analyzed using Stata 14. Two stage Data Envelopment Analysis (DEA) was performed. At the first stage technical efficiency (TE) scores were identified using DEA Program, version 2.1 (DEAP 2.1) developed by Tim Coelli [24]. In the second stage, the estimated TE scores of neonatal health service were used as dependent variables to assess its association with organizational and environmental factors. We fit a separate Tobit model for health posts and health centers. Significant independent determinants were identified at *p*-value less than 0.05, and 95% confidence interval of coefficients.

### DEA conceptual framework

DEA, a linear programming model, measures the relative performance of organizational units (in this case health centers and health posts) using multiple inputs and multiple outputs [25, 26].

$$ Technical\ Efficiency=\frac{Weighted\  sum\  of\ outputs}{Weighted\  sum\  of\ inputs} $$

#### Orientation

We used an input oriented TE measure, because decision makers can better influence inputs rather than outputs. This study aimed to give recommendation to woreda health offices and health centers that supervise health posts. The health centers supervising the health posts and the Woreda Health Offices are who make the decision about resource allocation in health centers and health posts. This measure addresses the question: “By how much can input quantities be proportionally reduced without changing the output quantities produced?” The choice of the approach is recommended to be based on which side of the orientation (input or outputs) the decision makers in the health facility have more control over [27].

### Model specification

There are constant and variable returns to scale models of measuring relative efficiency of decision-making units. The Constant Returns to Scale (CRS) model assumes a production process in which the optimal mix of inputs and outputs is independent of the scale of operation. TE scores obtained from a CRS DEA decomposed into two components, one due to scale inefficiency and one due to “pure” technical inefficiency [24, 28].

In a situation where health facilities are not operating at an optimal scale, the TE measure will be mixed with scale efficiency. Hence, to separate the two efficiency scores variable returns to scale (VRS) model is considered. VRS is an extension of equation of the CRS model after imposing a convexity constraint on it. This means that the data are enveloped more closely than the CRS model. The main advantage of this model is that it enables an inefficient health facility to be relatively compared with efficient health facilities of the same size only. Thus, the relative efficiency score of health facilities can be obtained by solving an equation as given by Cooper A. et al. [29]:

$$ Efficiency=\mathit{\operatorname{Max}}\sum \limits_r{U}_r{y}_{rjo}+{U}_0 $$

Subject to

$$ \sum \limits_r{U}_r{y}_{rj}-\sum \limits_r{V}_i{X}_{\mathrm{i}j}+{U}_0\le 0;\mathrm{j}=1,\dots, \mathrm{n} $$

$$ \sum \limits_i{V}_i{X}_{ijo}=1 $$

$$ {U}_r,{V}_i\ge 0 $$

Where:

Yrj = the amount of output r produced by health facility j,

Xij = the amount of input I used by health facility j,

Ur = the weight given to output r, (r = 1… t and t is the number of outputs),

Vi = the weight given to input I, (I = 1… m and m is the number of inputs),

j0 = the health facility under assessment

The study has employed VRS model of TE. VRS is preferred if the interest is on the extent to which the scale of operations affects productivity or when not all units of analysis are considered to be operating at an optimal scale. This is referred to as scale inefficiency and takes two forms – Decreasing Returns to Scale (DRS) and Increasing Returns to Scale (IRS). DRS imply that a health facility is too large for the volume of activities that it conducts. In order to operate at the most productive scale size, a health facility exhibiting DRS should scale down its scale of operation. In contrast, a health facility with IRS is too small for its scale of operation. If a health facility is exhibiting IRS, it should expand its scale of operation in order to become scale efficient [30, 31].

## Results

### Input and output variables

Under the primary health care unit (PHCU) neonatal health services are delivered at both health centers and health posts. We obtained complete information from 23 health centers and 68 health posts. These health care units utilized staff and other recurrent expenses for drugs and supplies to give neonatal outpatient and other service packages. Table 1 shows the descriptive statistics of the input and output variables used in the DEA model and stratified by “efficient” and “inefficient” health posts and health centers based on the technical efficiency scores produced in the DEA model, discussed in more detail below. The health posts spent a sum of US$ 24,503.65 for salary and US$ 1738.28 for non-salary recurrent expenditures to provide 8716 outpatient visits, 303 referrals and 3709 home visit services for neonates. Similarly, health centers spent US$ 33,899.62 for non-salary and US$ 53,214.25 for salary expenditures to render service for 1790 neonates at outpatient units, 17,728 at maternal and child health units and 165 neonatal referrals.

**Table 1:** Input for and output of neonatal health services among efficient and inefficient units, Southwest Ethiopia, 2018

Variables		Efficient			Inefficient		
		Mean	SD	Sum	Mean	SD	Sum
Health posts							
Input	HEW salary	208.13	339.70	1665.04	380.64	451.29	22,838.61
	Non-salary expense^a^	6.01	3.70	48.07	28.17	61.34	1690.21
Output	OPD	196	212.59	1570	119	160.76	7146
	Referral	13	16.44	101	3	3.29	202
	Home visit	100	112.28	798	49	87.03	2911
Health center							
Input	Non-salary expense^a^	687.21	955.13	5497.64	1893.47	2064.79	28,401.98
	Admin staff salary	397.02	592.65	3176.16	618.61	309.46	9279.13
	HO salary	289.46	409.28	2315.67	566.54	856.70	8498.15
	Nurse salary	650.65	885.20	5205.18	1198.84	1332.43	17,982.67
	Pharmacy salary	28.02	38.45	224.14	215.86	56.95	3237.96
	Lab salary	96.51	147.15	772.07	168.21	168.99	2523.12
Output	OPD	73	62.03	584	80	179.89	1206
	Referral	10	13	76	6	4.91	89
	MCH	516	594.16	4125	907	935.44	13,603

^a^expenses for vaccine, medicine and supplies

### Technical efficiency of neonatal health service

Only eight (11.76%) out of 68 health posts were operating efficiently in providing neonatal health services. The mean technical efficiency score of the health posts was 0.42 (± 0.30). This was the result of 17.65% pure technical and 11.76% scale efficiency (Additional file 1). Moreover, 63.24% were operating in an increasing returns to scale, implying they are too small for the scale of production. Expansion of scale of service improves the scale efficiency. In total, health posts could use US$ 14,577.92 per year to serve additional neonatal service demand if they were operating along the efficient frontier. This is almost 56% of the studied health posts total expenditure for neonatal health services (Table 2).

**Table 2:** Efficiency and potential cost saving of neonatal health services at primary health care facilities, Southwest Ethiopia, 2018

	Health Posts			Health centers						
		Potential saving			Potential saving					
	TE	HEWs salary	Recurrent expense^a^	TE	Recurrent expense^a^	Administrative staff	Health officers	Nurses	Pharmacy professionals	Laboratory professionals
Sum		13,133.99	1443.93		4928.70	2693.00	1333.40	6440.20	648.10	608.10
Mean	0.42	193.15	21.23	0.76	214.29	117.10	57.98	280.01	28.18	26.44
SD	0.30	323.85	56.96	0.26	420.87	325.90	136.94	809.66	76.37	50.57
Min	0.021	0.00	0.00	0.25	0.00	0.00	0.00	0.00	0.00	0.00
Max	1	1970.49	378.88	1.00	1640.78	1468.80	544.97	2943.57	327.74	149.04

The details are available as a Additional files 1 and 2

^a^expense for vaccine, medicine and supplies

On the other hand, eight (34.78%) health centers were found to be operating at technically efficient out of the 23 health centers. Mean technical efficiency score for the health centers was 0.75 (± 0.26). Out of the total health centers, 65.22% were pure technically efficient and 34.78% were scale efficient. Almost 44% of the health centers were operating in a decreasing returns to scale (Additional file 2). They should scale down their operation to become scale efficient. If they were operating efficiently as the efficient facilities, the inefficient health centers could use US$ 16,651.87 for additional services. This is more than 19% of the total spending of health centers for neonatal health services (Table 2).

**Fig. 1:**
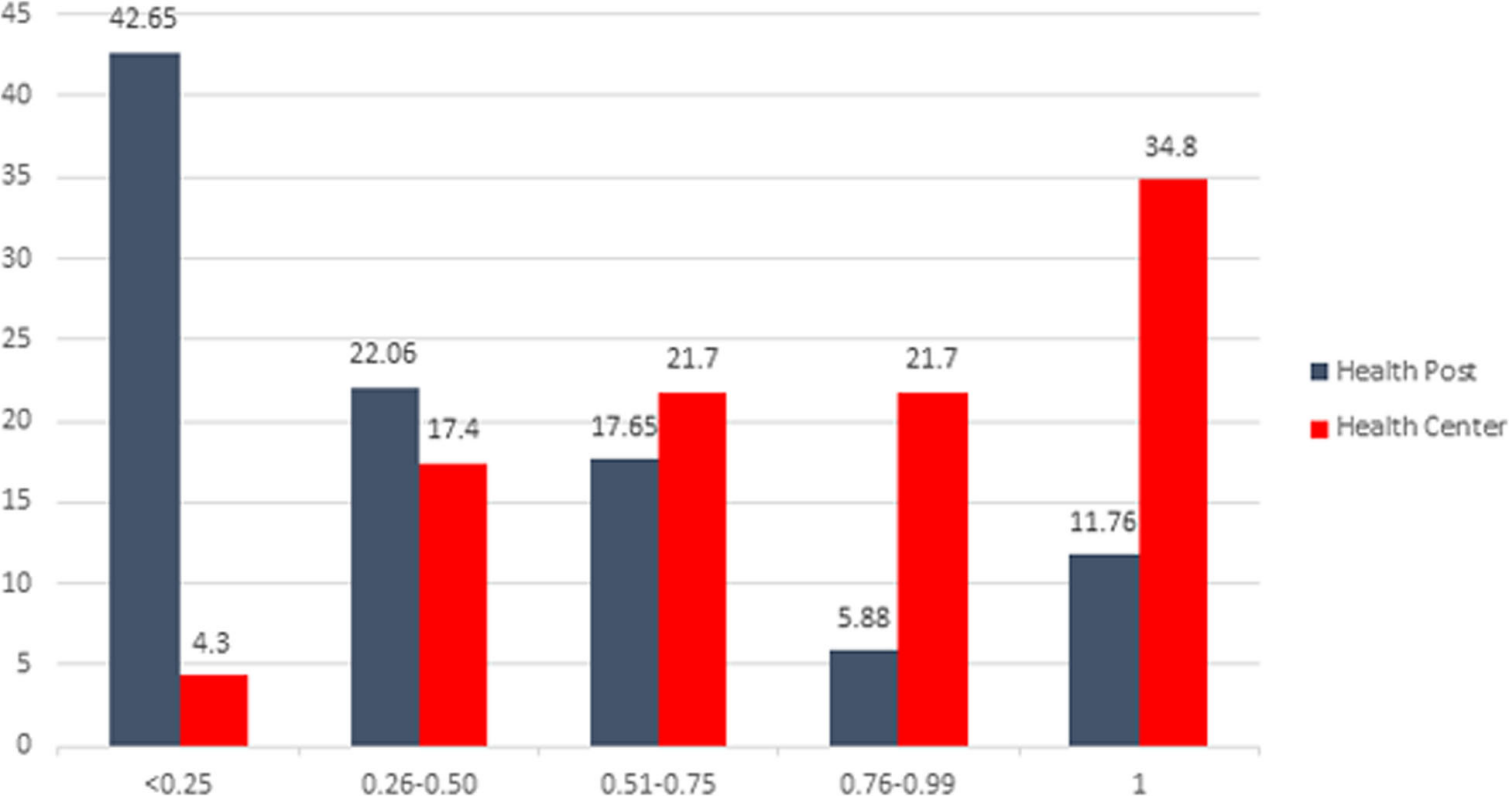
Distribution of neonatal health services’ technical efficiency scores among health posts and health centers Southwest Ethiopia, 2018

The highest proportion (42.65%) of health posts’ efficiency score was concentrated between 0 and 0.25. On the contrary, the lowest proportion (5.88%) were in between 0.76 and 0.99. On the other hand, highest proportion (34.8%) of health centers were fount technically efficient, only 4.3% were least efficient (Fig. 1).

### Determinants of neonatal health services’ technical efficiency in the primary health care

The results of the Tobit regression model for examining determinants of technical efficiency are given in Table 3. Health centers technical efficiency scores was positively affected both by the health centers head’s years of experience [β = 0.013, 95% CI, − 0.001, 0.026] and catchment population of the facility [β = 0.00002, 95% CI, 2.13E-06, 2.98E-05]. For health posts, the total number of neonates in the catchment population [β = 0.0006, 95% CI, 0.0002, 0.001] positively affected the technical efficiency scores, while waiting time for neonatal health service [β = − 0.01, 95% CI, − 0.018, − 0.003] was negatively associated with the technical efficiency scores in health posts.

**Table 3:** Determinants of technical efficiency of neonatal health services in health centers and health posts, Southwest Ethiopia, 2018

Variables	Health center		Health post	
	Coef. (95% CI)	*P*	Coef. (95% CI)	*P*
Service years of the facility	−0.006 (− 0.018, 0.006)	0.29	− 0.0169 (− 0.047, 0.013)	0.27
Facility head’s years of experience	0.013 (− 0.001, 0.026)	0.06^*^		
Catchment population	< 0.001 (2.13E-06, 2.98E-05)	0.03^**^	−1.33E-06 (− 3.6E-05, 3.36E-05)	0.94
Availability of health facility around	0.068 (−0.26, 0.395)	0.67	−0.01 (− 0.16, 0.15)	0.92
Facility head’s level of education	−0.163 (− 0.5, 0.174)	0.32		
Neonates in the catchment population			0.0006 (0.0002, 0.001)	< 0.01^**^
Waiting time for service			−0.01 (−0.018, − 0.003)	< 0.01^**^
_cons	0.244 (−0.222, 0.711)	0	0.749 (0.383, 1.114)	
/sigma	0.294 (0.172, 0.415)		0.29 (0.383, 0.345)	

^*^significant at *p* < 0.1, ^**^significant at *p* < 0.05

## Discussion

Good performance of the health system is imperative in order to improve the overall health status and contribution of citizens towards the development of a country. Performance by itself is an abstract and complex concept and can be measured through several dimensions. Different countries with similar economic status may differ in their health outcome because of difference in performance [32, 33]. In economic terms, effectiveness, efficiency and equity can be used to measure health systems’ performance [34, 35]. In this analysis, we used efficiency to measure performance.

In relation to neonatal health service in Ethiopia we focused on four major outputs on the primary health care system: neonatal outpatient service, maternal and child health service, referral services and community-based home-to-home services for newborns. Public health facilities in the study area were spending resources on various inputs ranging from human resources to medical supplies, so as to render these services to newborns. Relative measure of technical efficiency was employed to identify the proportion of health service units that operate efficiently and inefficiently by health facility type.

The result of our study revealed that there is variation on the relative use of human and other resources for providing neonatal services across health posts and health centers. The proportion of inefficient health posts is higher than the health centers, 88.24% versus 65.2% respectively. On average, health posts could have saved 58% of their inputs without altering the existing outputs. Similarly, health centers could have saved 25% of their resources without reducing outputs. While previous studies haven’t specifically measured efficiency of neonatal health services, they have measured technical efficiency of units in the primary health care system and reported similar findings to ours. For instance, studies in Ethiopia on health posts and health centers found 75% [15] and 50% [14] technical inefficiency, respectively. Studies from Kenya conversely reported a much lower level of inefficiency, 44% [36]. The result from three studies conducted in Ghanaian health centers is almost in line with our study, 69% [37], 65% [28] and 78% [38] technical inefficiency.

The range of technical efficiency scores in health posts was wide, from 0.02 to 1.00. This implies that there is huge discrepancy in how resources are used among health posts in the operation of neonatal health services. On the other hand, the efficiency of health centers range between 0.25 and 1.00. Although the efficiency variation among the health centers is narrower, there is still a wide gap (75%) in resource utilization. In a similar study done in the United Kingdom, the efficiency disparity along health care delivery units was almost the same as what we found for health centers. Efficiency among neonatal health delivery units ranged from 0.24 to 1.00, which is almost 74% [20].

As we go through the outputs of both health posts and health centers, we come across with discrepancies between efficient and inefficient units. For instance there are far fewer home visits in the inefficient health posts compared to the efficient ones. Perhaps there needs to be more supervision from the health centers to make sure that HEWs are performing their roles. In the case of health centers, there are higher average outputs for the inefficient health centers (with exception of referrals) compared to the efficient health centers. So this brings into question whether the inefficient health centers should really reduce their inputs or if the efficient health centers are operating at a lower level – low staff and low outputs? This might not be very effective in health service provision, even though it looks “efficient”.

The technical efficiency scores of nearly half of health posts are between 0 and 0.25. Contrary to the case of the health posts, efficiency score distribution for the largest share of health centers (34.8%) were found 100% technically efficient. Only 4.3% are with less than 0.25 efficiency score. This may perhaps happened because most of mothers tend to use neonatal health services at health center or hospitals they delivered, since some of the services given during postnatal period. For the fact that delivery cases have not being managed in a health post, low number of neonatal output can be exhibited in the health posts as compared to health centers.

We found that inefficient health posts could save US$ 14,577.92 per year, if they were operating efficiently. To put this in perspective, when we compare this with the Ethiopian per capita health expenditure, US$ 28.65 as indicated in the sixth national health accounts [39], these funds would provide a year’s worth of health care for 509 people. Even if we see it with World Health Organization’s (WHO’s) recommendation per capita for low and middle income countries (US$ 60), it worth the yearly expenditure of 243 people [40]. Similarly, in health centers resources equivalent to US$ 16,651.87 were spent inefficiently. This amounts to a year’s worth of health expenditure for 581 and 278 according to the Ethiopian national per capita health expenditure [39] and WHO’s recommendation [40], respectively.

The technical efficiency of neonatal health services in the primary health care system of southwest Ethiopia was associated with a number of factors. In health posts, efficiency was associated positively with the number of neonates in the catchment population and negatively with length of waiting time for neonatal health services. An increase in one neonate in the catchment population resulted in 0.06% increase in technical efficiency score. A one-minute increase in waiting time on the other hand resulted in 1.03% decrease in technical efficiency score. A study conducted in Tigray, Ethiopia [15], didn’t report any factors significantly associated with technical efficiency of health posts. In that study the authors fitted a regression model to examine for association between technical efficiency of health posts (which is organizational) with health extension workers characteristics (which is individual), rather than other organizational and environmental determinants.

Similarly, technical efficiency of neonatal health services in health centers was positively associated with two variables, the size of the catchment population of the facility and health centers head’s years of experience. A one-year increase in the health center head’s experience resulted in 1.3% increase in the efficiency score. Moreover, a unit increase in the catchment population of the facility resulted in 0.002% increase in technical efficiency of health centers in delivering neonatal health services. A study in Ghana also reported service years of the health center having a positive effect on the technical efficiency of health centers [38]. However, this variable was not among the determinants of efficiency in our study. In line with our study, Bobo et al. [14] have found that catchment population of the facility affected the technical efficiency of health centers. However, the regression model of study by Bobo et al. included variables used to estimate the technical efficiency scores as explanatory variables, and their model may suffer from autocorrelation.

There were some limitations in this study. First, health service utilization has direct effect on technical efficiency of health facilities. The study did not investigate social, cultural and behavioral factors, which can strongly influence the outputs of health systems. Second, to get specific estimates of neonatal health service inputs we had to use estimates the general health facility resources proportionally. This might have resulted in under or over estimation of the inputs used to provide neonatal health services. This proportional allocation forced us to measure resources in terms of money rather than the resources as they are. Finally, the study has not considered fixed costs, theoretically that could have introduced bias.

## Conclusion

Generally, the inefficiency of neonatal health service delivery in the primary health care facilities of Southwest Ethiopia is high. It is much worse in health posts than health centers. The additional salary as well as non-salary recurrent expenditures could be used for additional neonatal health services or for any other service.

The technical efficiency of health service delivery units was significantly affected by catchment population, experience of the head of facility and waiting time for neonatal health services. Moreover, performance improvement measures targeted at optimal use of available resources are recommended. Providing more adequate training to heads of health facilities and improving staffs time management in order to reduce waiting times for neonatal health services are perhaps the most financially feasible interventions for effective efficiency gains.

## Supplementary information

**Additional file 1: Table S1.** Technical efficiency and cost of neonatal health services at health postsand health centers Southwest Ethiopia, 2018.

**Additional file 2: **Potential input reduction health posts and health centers, Southwest Ethiopia, 2018.

**Additional file 3: **Data used in the study, Southwest Ethiopia, 2018.

## Data Availability

All data generated or analyzed during this study are included in this published article [Additional file 3].

## Abbreviations

CBNC: Community based newborn care
CRS: Constant Returns to Scale
DRS: Decreasing Returns to Scale
ETB: Ethiopian birr
HEWs: Health Extension Workers
HO: Health Officer
IMNCI: Integrated management of newborn and child illness
IRS: Increasing Returns to Scale
MCH: Maternal and Child Health
NICU: Neonatal intensive care unit
OPD: Outpatient Department.
PHCU: Primary health care unit
SDG: Sustainable Development Goal
TE: Technical efficiency
US$: United States dollar
VRS: Variable returns to scale
WHO: World Health Organization
