# Supplementary material for: Technical efficiency of neonatal health services in primary health care facilities of Southwest Ethiopia: a two-stage data envelopment analysis
Source: Health Econ Rev. 2019 Oct 27;9:27. doi: 10.1186/s13561-019-0245-7 (PMC6815357; doi:10.1186/s13561-019-0245-7)
Supplement: Supplementary file 1 — Additional file 1: Table S1. Technical efficiency and cost of neonatal health services at health postsand health centers Southwest Ethiopia, 2018. [file 13561_2019_245_MOESM1_ESM.docx]

**Additional file 1**

**Table S1: Efficiency and cost of neonatal health services at health posts, Southwest Ethiopia, 2018**

| **Unit** | **CRS_TE** | **VRS_TE** | **Scale Efficiency** | **Economies of scale** | **Total cost ($)** | **Adjusted total cost ($)** | **Potential cost saving ($)** |
| --- | --- | --- | --- | --- | --- | --- | --- |
| HP01 | 0.21 | 0.307 | 0.684 | drs | 2252.62 | 271.08 | 1981.53 |
| HP02 | 1 | 1 | 1 | - | 1028.22 | 1028.22 | 0.00 |
| HP03 | 0.96 | 0.962 | 0.998 | irs | 591.53 | 569.02 | 22.51 |
| HP04 | 0.399 | 0.41 | 0.971 | irs | 914.97 | 375.55 | 539.43 |
| HP05 | 0.366 | 0.528 | 0.694 | irs | 30.95 | 16.34 | 14.61 |
| HP06 | 0.219 | 0.23 | 0.953 | irs | 94.70 | 21.75 | 72.95 |
| HP07 | 0.191 | 0.234 | 0.815 | irs | 109.02 | 25.56 | 83.46 |
| HP08 | 0.111 | 0.179 | 0.62 | drs | 530.32 | 70.28 | 460.04 |
| HP09 | 0.083 | 0.119 | 0.7 | drs | 431.45 | 40.49 | 390.95 |
| HP10 | 0.071 | 0.106 | 0.671 | drs | 529.60 | 43.63 | 485.97 |
| HP11 | 0.178 | 0.203 | 0.879 | irs | 163.72 | 33.16 | 130.56 |
| HP12 | 0.728 | 0.794 | 0.917 | drs | 52.25 | 34.56 | 17.69 |
| HP13 | 0.206 | 0.21 | 0.981 | irs | 85.61 | 17.98 | 67.62 |
| HP14 | 1 | 1 | 1 | - | 124.34 | 124.34 | 0.00 |
| HP15 | 0.226 | 0.264 | 0.855 | drs | 1428.55 | 377.61 | 1050.94 |
| HP16 | 0.822 | 0.838 | 0.981 | irs | 203.59 | 170.70 | 32.88 |
| HP17 | 0.575 | 0.59 | 0.973 | irs | 482.49 | 284.88 | 197.61 |
| HP18 | 0.439 | 0.441 | 0.995 | irs | 1342.49 | 592.22 | 750.28 |
| HP19 | 1 | 1 | 1 | - | 207.11 | 207.11 | 0.00 |
| HP20 | 0.228 | 0.266 | 0.858 | irs | 135.77 | 36.11 | 99.67 |
| HP21 | 0.591 | 0.603 | 0.98 | irs | 256.44 | 154.74 | 101.70 |
| HP22 | 0.281 | 0.342 | 0.822 | irs | 63.13 | 21.57 | 41.56 |
| HP23 | 0.521 | 0.566 | 0.921 | irs | 55.90 | 31.64 | 24.26 |
| HP24 | 0.324 | 0.328 | 0.988 | drs | 111.47 | 35.53 | 75.94 |
| HP25 | 0.541 | 0.622 | 0.87 | drs | 59.53 | 34.62 | 24.91 |
| HP26 | 0.36 | 0.362 | 0.995 | drs | 216.93 | 78.56 | 138.37 |
| HP27 | 0.368 | 0.37 | 0.996 | drs | 186.94 | 69.07 | 117.86 |
| HP28 | 0.289 | 0.299 | 0.965 | irs | 922.22 | 275.99 | 646.23 |
| HP29 | 0.556 | 0.646 | 0.861 | irs | 66.36 | 42.90 | 23.47 |
| HP30 | 0.099 | 1 | 0.099 | irs | 19.26 | 19.26 | 0.00 |
| HP31 | 0.071 | 0.09 | 0.789 | drs | 828.13 | 48.12 | 780.01 |
| HP32 | 0.144 | 0.199 | 0.725 | irs | 138.02 | 27.46 | 110.56 |
| HP33 | 0.021 | 0.042 | 0.485 | irs | 492.08 | 20.89 | 471.19 |
| HP34 | 0.135 | 0.14 | 0.964 | irs | 394.14 | 55.23 | 338.92 |
| HP35 | 0.254 | 0.269 | 0.945 | irs | 217.58 | 58.56 | 159.02 |
| HP36 | 0.385 | 0.413 | 0.933 | drs | 94.79 | 34.01 | 60.78 |
| HP37 | 0.446 | 0.449 | 0.993 | irs | 111.79 | 50.17 | 61.61 |
| HP38 | 0.509 | 0.568 | 0.897 | irs | 29.78 | 16.91 | 12.87 |
| HP39 | 0.227 | 0.268 | 0.847 | irs | 52.11 | 13.97 | 38.14 |
| HP40 | 0.174 | 0.284 | 0.614 | irs | 85.35 | 24.25 | 61.10 |
| HP41 | 0.16 | 0.193 | 0.833 | irs | 76.50 | 14.73 | 61.77 |
| HP42 | 1 | 1 | 1 | - | 18.46 | 18.46 | 0.00 |
| HP43 | 0.184 | 0.203 | 0.905 | irs | 453.01 | 91.86 | 361.15 |
| HP44 | 0.496 | 0.497 | 0.997 | irs | 111.64 | 55.53 | 56.12 |
| HP45 | 1 | 1 | 1 | - | 194.64 | 194.64 | 0.00 |
| HP46 | 0.359 | 0.406 | 0.883 | irs | 129.06 | 52.42 | 76.65 |
| HP47 | 0.551 | 0.568 | 0.972 | irs | 233.02 | 132.27 | 100.75 |
| HP48 | 0.48 | 0.716 | 0.671 | drs | 473.81 | 339.42 | 134.39 |
| HP49 | 1 | 1 | 1 | - | 125.14 | 125.14 | 0.00 |
| HP50 | 0.913 | 1 | 0.913 | drs | 1338.07 | 1338.07 | 0.00 |
| HP51 | 0.736 | 0.793 | 0.928 | drs | 1062.63 | 843.01 | 219.62 |
| HP52 | 0.121 | 0.19 | 0.637 | irs | 71.00 | 13.48 | 57.52 |
| HP53 | 1 | 1 | 1 | - | 8.36 | 8.36 | 0.00 |
| HP54 | 1 | 1 | 1 | - | 6.83 | 6.83 | 0.00 |
| HP55 | 0.661 | 0.816 | 0.81 | irs | 16.02 | 13.08 | 2.94 |
| HP56 | 0.405 | 0.637 | 0.636 | irs | 250.25 | 159.50 | 90.75 |
| HP57 | 0.302 | 0.45 | 0.671 | irs | 441.64 | 198.53 | 243.11 |
| HP58 | 0.18 | 0.211 | 0.854 | irs | 112.16 | 23.66 | 88.50 |
| HP59 | 0.205 | 0.223 | 0.919 | irs | 83.45 | 18.63 | 64.82 |
| HP60 | 0.773 | 1 | 0.773 | irs | 135.29 | 135.29 | 0.00 |
| HP61 | 0.059 | 0.059 | 0.998 | - | 609.67 | 35.93 | 573.74 |
| HP62 | 0.102 | 0.105 | 0.971 | irs | 659.51 | 69.33 | 590.18 |
| HP63 | 0.088 | 0.088 | 0.993 | irs | 1288.95 | 113.78 | 1175.16 |
| HP64 | 0.231 | 0.401 | 0.575 | irs | 1067.62 | 428.51 | 639.11 |
| HP65 | 0.55 | 0.917 | 0.6 | irs | 767.26 | 703.24 | 64.01 |
| HP66 | 0.657 | 1 | 0.657 | irs | 992.70 | 992.70 | 0.00 |
| HP67 | 0.205 | 0.215 | 0.952 | irs | 262.30 | 56.41 | 205.89 |
| HP68 | 0.138 | 0.252 | 0.549 | drs | 111.71 | 27.17 | 84.54 |
| Sum |  |  |  |  | 26241.93 | 11664.00 | 14577.93 |
| mean | 0.42 | 0.49 | 0.85 |  | 385.91 | 171.53 | 214.38 |
| SD | 0.30 | 0.32 | 0.17 |  | 448.16 | 269.76 | 339.76 |
| Minimum | 0.021 | 0.04 | 0.10 |  | 6.83 | 6.83 | 0.00 |
| Maximum | 1 | 1 | 1 |  | 2252.616 | 1338.07 | 1981.532 |

**Table S2: Efficiency and cost of neonatal health services at health centers, Southwest Ethiopia, 2018**

| **Unit** | **CRS_TE** | **VRS_TE** | **Scale Efficiency** | **Economies of scale** | **Total cost ($)** | **Adjusted total cost ($)** | **Potential cost saving ($)** |
| --- | --- | --- | --- | --- | --- | --- | --- |
| HC01 | 0.248 | 0.396 | 0.628 | drs | 7978.03 | 2446.97 | 5531.06 |
| HC02 | 1.000 | 1.000 | 1.000 |  | 2370.94 | 2370.94 | 0 |
| HC03 | 0.793 | 1.000 | 0.793 | drs | 2609.83 | 2609.83 | 0 |
| HC04 | 1.000 | 1.000 | 1.000 |  | 981.977 | 981.977 | 0 |
| HC05 | 0.665 | 0.678 | 0.981 | irs | 871.657 | 377.002 | 494.655 |
| HC06 | 0.632 | 1.000 | 0.632 | drs | 12654.5 | 12654.5 | 0 |
| HC07 | 0.505 | 1.000 | 0.505 | drs | 15348.9 | 15348.9 | 0 |
| HC08 | 0.375 | 0.722 | 0.519 | drs | 11609.5 | 5370.8 | 6238.74 |
| HC09 | 0.963 | 1.000 | 0.963 | irs | 477.066 | 477.066 | 0 |
| HC10 | 0.490 | 0.625 | 0.784 | irs | 939.492 | 521.757 | 417.734 |
| HC11 | 1.000 | 1.000 | 1.000 |  | 5990.05 | 5990.05 | 0 |
| HC12 | 0.593 | 0.867 | 0.684 | drs | 1742.62 | 1244.58 | 498.037 |
| HC13 | 0.305 | 0.513 | 0.594 | drs | 3815.59 | 1443.42 | 2372.18 |
| HC14 | 1.000 | 1.000 | 1.000 |  | 156.551 | 156.551 | 0 |
| HC15 | 1.000 | 1.000 | 1.000 |  | 4543.72 | 4543.72 | 0 |
| HC16 | 0.988 | 1.000 | 0.988 | irs | 723.789 | 723.788 | 0.00037 |
| HC17 | 0.804 | 1.000 | 0.804 | drs | 4665.32 | 4665.32 | 0 |
| HC18 | 0.861 | 1.000 | 0.861 | drs | 4605.5 | 4605.5 | 0 |
| HC19 | 0.443 | 0.450 | 0.985 | irs | 698.392 | 262.56 | 435.832 |
| HC20 | 1.000 | 1.000 | 1.000 |  | 661.999 | 661.999 | 0 |
| HC21 | 1.000 | 1.000 | 1.000 |  | 1545.78 | 1545.78 | 0 |
| HC22 | 1.000 | 1.000 | 1.000 |  | 939.847 | 939.847 | 0 |
| HC23 | 0.698 | 0.733 | 0.952 | drs | 1182.73 | 519.102 | 663.632 |
| Sum |  |  |  |  | 87113.88 | 70462.01 | 16651.87 |
| Mean | 0.76 | 0.87 | 0.86 |  | 3787.56 | 3063.57 | 723.99 |
| SD | 0.26 | 0.20 | 0.18 |  | 4278.47 | 3910.73 | 1710.71 |
| Min | 0.25 | 0.40 | 0.51 |  | 156.55 | 156.55 | 0.00 |
| Max | 1.00 | 1.00 | 1.00 |  | 15348.90 | 15348.90 | 6238.74 |
