# Supplementary material for: Technical efficiency of neonatal health services in primary health care facilities of Southwest Ethiopia: a two-stage data envelopment analysis
Source: Health Econ Rev. 2019 Oct 27;9:27. doi: 10.1186/s13561-019-0245-7 (PMC6815357; doi:10.1186/s13561-019-0245-7)
Supplement: Supplementary file 2 — Additional file 2: Potential input reduction health posts and health centers, Southwest Ethiopia, 2018. [file 13561_2019_245_MOESM2_ESM.docx]

**Additional file 2: Potential input reduction**

**Table S1: Health posts’ potential input reduction**

| **Firm input** | **HEWs salary expense** | | **Non-salary recurrent expense** | |
| --- | --- | --- | --- | --- |
|  | **Targets** | **Potential cost saving** | **Targets** | **Potential cost saving** |
| HP01 | 266.20 | 1970.49 | 4.88 | 11.04 |
| HP02 | 1025.70 | 0.00 | 2.52 | 0.00 |
| HP03 | 564.88 | 22.34 | 4.15 | 0.16 |
| HP04 | 373.48 | 536.45 | 2.07 | 2.97 |
| HP05 | 14.80 | 13.22 | 1.55 | 1.39 |
| HP06 | 19.76 | 66.28 | 1.99 | 6.67 |
| HP07 | 23.54 | 76.88 | 2.02 | 6.58 |
| HP08 | 60.71 | 279.05 | 9.56 | 180.99 |
| HP09 | 33.19 | 246.16 | 7.30 | 144.79 |
| HP10 | 36.09 | 305.12 | 7.54 | 180.85 |
| HP11 | 30.91 | 121.67 | 2.26 | 8.89 |
| HP12 | 28.96 | 7.51 | 5.59 | 10.19 |
| HP13 | 13.34 | 50.15 | 4.65 | 17.48 |
| HP14 | 118.52 | 0.00 | 5.82 | 0.00 |
| HP15 | 374.10 | 1041.18 | 3.51 | 9.76 |
| HP16 | 166.11 | 32.00 | 4.60 | 0.88 |
| HP17 | 281.82 | 195.48 | 3.06 | 2.12 |
| HP18 | 588.71 | 745.82 | 3.51 | 4.45 |
| HP19 | 200.97 | 0.00 | 6.14 | 0.00 |
| HP20 | 33.77 | 93.23 | 2.33 | 6.43 |
| HP21 | 150.11 | 98.66 | 4.63 | 3.04 |
| HP22 | 19.33 | 37.24 | 2.24 | 4.32 |
| HP23 | 27.35 | 20.98 | 4.29 | 3.29 |
| HP24 | 31.33 | 64.30 | 4.20 | 11.64 |
| HP25 | 27.76 | 16.85 | 6.85 | 8.06 |
| HP26 | 69.00 | 121.52 | 9.57 | 16.85 |
| HP27 | 60.66 | 103.51 | 8.41 | 14.35 |
| HP28 | 273.44 | 640.27 | 2.55 | 5.96 |
| HP29 | 40.89 | 22.37 | 2.00 | 1.10 |
| HP30 | 18.27 | 0.00 | 0.99 | 0.00 |
| HP31 | 39.50 | 401.12 | 8.62 | 378.88 |
| HP32 | 25.58 | 102.97 | 1.88 | 7.59 |
| HP33 | 19.75 | 445.32 | 1.15 | 25.87 |
| HP34 | 51.18 | 314.11 | 4.04 | 24.81 |
| HP35 | 54.31 | 147.50 | 4.24 | 11.52 |
| HP36 | 30.82 | 43.78 | 3.20 | 17.00 |
| HP37 | 45.33 | 55.66 | 4.84 | 5.95 |
| HP38 | 13.48 | 10.26 | 3.42 | 2.60 |
| HP39 | 11.83 | 32.29 | 2.15 | 5.86 |
| HP40 | 22.95 | 57.83 | 1.30 | 3.27 |
| HP41 | 13.13 | 55.05 | 1.60 | 6.72 |
| HP42 | 11.78 | 0.00 | 6.68 | 0.00 |
| HP43 | 89.35 | 351.27 | 2.51 | 9.89 |
| HP44 | 51.85 | 52.41 | 3.68 | 3.71 |
| HP45 | 187.71 | 0.00 | 6.93 | 0.00 |
| HP46 | 49.95 | 73.05 | 2.46 | 3.60 |
| HP47 | 128.12 | 97.59 | 4.15 | 3.16 |
| HP48 | 332.89 | 131.80 | 6.53 | 2.58 |
| HP49 | 111.41 | 0.00 | 13.73 | 0.00 |
| HP50 | 1319.08 | 0.00 | 18.99 | 0.00 |
| HP51 | 832.93 | 216.99 | 10.08 | 2.63 |
| HP52 | 12.13 | 51.78 | 1.35 | 5.74 |
| HP53 | 3.50 | 0.00 | 4.86 | 0.00 |
| HP54 | 5.44 | 0.00 | 1.39 | 0.00 |
| HP55 | 8.06 | 1.82 | 5.01 | 1.13 |
| HP56 | 158.24 | 90.03 | 1.26 | 0.72 |
| HP57 | 197.20 | 241.48 | 1.33 | 1.63 |
| HP58 | 21.32 | 79.75 | 2.34 | 8.75 |
| HP59 | 15.61 | 54.31 | 3.02 | 10.50 |
| HP60 | 133.90 | 0.00 | 1.39 | 0.00 |
| HP61 | 31.36 | 500.85 | 4.56 | 72.90 |
| HP62 | 62.44 | 531.53 | 6.89 | 58.65 |
| HP63 | 107.06 | 1105.69 | 6.73 | 69.47 |
| HP64 | 427.33 | 637.34 | 1.19 | 1.77 |
| HP65 | 702.31 | 63.93 | 0.94 | 0.09 |
| HP66 | 992.05 | 0.00 | 0.66 | 0.00 |
| HP67 | 51.43 | 187.72 | 4.98 | 18.17 |
| HP68 | 23.65 | 70.02 | 3.51 | 14.52 |
|  |  |  |  |  |
| Sum | 11369.66 | 13133.99 | 294.35 | 1443.93 |
| Mean | 167.20 | 193.15 | 4.33 | 21.23 |
| SD | 268.94 | 323.85 | 3.20 | 56.96 |
| Min | 3.50 | 0.00 | 0.66 | 0.00 |
| Max | 1319.08 | 1970.49 | 18.99 | 378.88 |

**Table S2: Health centers’ potential input reduction**

| **Health centers** | **Non-salary recurrent expenditure** | | **Administrative staff salary expense** | | **Health officers’ salary expense** | | **Nurses’ salary expense** | | **Pharmacy professionals salary expense** | | **Laboratory professionals’ salary expense** | |
| --- | --- | --- | --- | --- | --- | --- | --- | --- | --- | --- | --- | --- |
|  | **Targets** | **Potential saving** | **Targets** | **Potential saving** | **Targets** | **Potential saving** | **Targets** | **Potential saving** | **Targets** | **Potential saving** | **Targets** | **Potential saving** |
| HC01 | 1073.59 | 1640.78 | 319.51 | 488.31 | 252.76 | 386.30 | 606.07 | 2717.58 | 97.52 | 149.04 | 97.52 | 149.04 |
| HC02 | 322.35 | 0.00 | 439.54 | 0.00 | 425.83 | 0.00 | 757.38 | 0.00 | 0.00 | 0.00 | 425.83 | 0.00 |
| HC03 | 1286.54 | 0.00 | 309.46 | 0.00 | 337.31 | 0.00 | 554.58 | 0.00 | 56.95 | 0.00 | 64.99 | 0.00 |
| HC04 | 360.69 | 0.00 | 117.88 | 0.00 | 136.02 | 0.00 | 278.40 | 0.00 | 17.56 | 0.00 | 71.42 | 0.00 |
| HC05 | 182.66 | 336.70 | 39.88 | 18.93 | 49.82 | 39.96 | 76.58 | 85.74 | 12.21 | 5.80 | 15.86 | 7.53 |
| HC06 | 7026.01 | 0.00 | 1492.81 | 0.00 | 855.41 | 0.00 | 2456.37 | 0.00 | 634.23 | 0.00 | 189.70 | 0.00 |
| HC07 | 5576.41 | 0.00 | 1939.72 | 0.00 | 3390.03 | 0.00 | 2983.74 | 0.00 | 824.83 | 0.00 | 634.19 | 0.00 |
| HC08 | 2657.59 | 1023.36 | 470.89 | 1468.80 | 709.09 | 544.97 | 1007.70 | 2943.57 | 263.24 | 128.53 | 262.28 | 129.49 |
| HC09 | 79.63 | 0.00 | 49.34 | 0.00 | 89.60 | 0.00 | 211.47 | 0.00 | 17.24 | 0.00 | 29.78 | 0.00 |
| HC10 | 239.23 | 143.26 | 61.97 | 95.27 | 74.52 | 44.63 | 113.54 | 115.12 | 10.73 | 6.42 | 21.77 | 13.04 |
| HC11 | 196.49 | 0.00 | 1775.30 | 0.00 | 1252.49 | 0.00 | 2765.76 | 0.00 | 0.00 | 0.00 | 0.00 | 0.00 |
| HC12 | 536.40 | 330.44 | 172.00 | 85.56 | 127.37 | 19.49 | 314.05 | 48.05 | 47.38 | 7.25 | 47.38 | 7.25 |
| HC13 | 590.82 | 883.07 | 138.08 | 488.29 | 188.02 | 178.51 | 389.43 | 369.73 | 64.49 | 327.74 | 72.57 | 124.84 |
| HC14 | 82.63 | 0.00 | 18.15 | 0.00 | 20.52 | 0.00 | 18.04 | 0.00 | 10.39 | 0.00 | 6.83 | 0.00 |
| HC15 | 2982.89 | 0.00 | 579.44 | 0.00 | 135.56 | 0.00 | 556.71 | 0.00 | 104.41 | 0.00 | 184.71 | 0.00 |
| HC16 | 375.04 | 0.00 | 148.28 | 0.00 | 29.28 | 0.00 | 117.22 | 0.00 | 34.18 | 0.00 | 19.80 | 0.00 |
| HC17 | 1259.66 | 0.00 | 1013.15 | 0.00 | 268.98 | 0.00 | 1698.76 | 0.00 | 268.98 | 0.00 | 155.80 | 0.00 |
| HC18 | 2196.71 | 0.00 | 362.01 | 0.00 | 697.16 | 0.00 | 853.73 | 0.00 | 224.06 | 0.00 | 271.83 | 0.00 |
| HC19 | 130.45 | 167.84 | 27.07 | 33.08 | 32.44 | 41.77 | 49.91 | 119.42 | 12.89 | 15.75 | 9.80 | 57.97 |
| HC20 | 385.68 | 0.00 | 30.19 | 0.00 | 58.45 | 0.00 | 155.37 | 0.00 | 22.42 | 0.00 | 9.89 | 0.00 |
| HC21 | 863.80 | 0.00 | 125.05 | 0.00 | 217.44 | 0.00 | 266.11 | 0.00 | 0.00 | 0.00 | 73.40 | 0.00 |
| HC22 | 303.11 | 0.00 | 90.62 | 0.00 | 69.35 | 0.00 | 407.41 | 0.00 | 69.35 | 0.00 | 0.00 | 0.00 |
| HC23 | 262.59 | 403.20 | 41.57 | 15.15 | 62.92 | 77.78 | 109.33 | 40.97 | 20.90 | 7.62 | 21.79 | 118.92 |
|  |  |  |  |  |  |  |  |  |  |  |  |  |
| Sum | 28971.00 | 4928.70 | 9761.89 | 2693.00 | 9480.41 | 1333.40 | 16747.66 | 6440.20 | 2813.96 | 648.10 | 2687.12 | 608.10 |
| Mean | 1259.60 | 214.29 | 424.43 | 117.10 | 412.19 | 57.98 | 728.16 | 280.01 | 122.35 | 28.18 | 116.83 | 26.44 |
| SD | 1801.20 | 420.87 | 573.09 | 325.90 | 722.10 | 136.94 | 885.75 | 809.66 | 209.79 | 76.37 | 156.83 | 50.57 |
| Min | 79.63 | 0.00 | 18.15 | 0.00 | 20.52 | 0.00 | 18.04 | 0.00 | 0.00 | 0.00 | 0.00 | 0.00 |
| Max | 7026.01 | 1640.78 | 1939.72 | 1468.80 | 3390.03 | 544.97 | 2983.74 | 2943.57 | 824.83 | 327.74 | 634.19 | 149.04 |
